# Hypoxia Supports LPS-Driven Tolerance and Functional Activation in BV-2 Microglial Cells

**DOI:** 10.3390/biology14111512

**Published:** 2025-10-28

**Authors:** Alicia Chavero Vargas, Natascha Köstlin-Gille, Reinhard Bauer, Stefanie Dietz-Ziegler, Anita S. Lokaj, Soumya Lutterbach, Christian Gille, Trim Lajqi

**Affiliations:** 1 Department of Neonatology, Medical Faculty Heidelberg, University of Heidelberg, 69120 Heidelberg, Germany; 2 Department of Neonatology, University of Tübingen, 72076 Tübingen, Germany; 3 Institute of Molecular Cell Biology, Jena University Hospital, 07745 Jena, Germany; 4 Department of Ophthalmology, University Center Clinic of Kosovo, XK-10000 Prishtina, Kosovo; 5 Department of Anesthesiology, Medical Faculty Heidelberg, University of Heidelberg, 69120 Heidelberg, Germany

**Keywords:** microglia, tolerance, normoxia, hypoxia, inflammation, migration, phagocytosis

## Abstract

**Simple Summary:**

This study explores the effects of short-term hypoxia on BV-2 microglial cells in vitro, focusing on inflammation, metabolism, and cellular functions. Our results show that hypoxia promotes a pronounced tolerant phenotype in BV-2 cells, characterized by reduced pro-inflammatory markers and decreased glycolytic activity via the MyD88/NF-κB p65 pathway. Tolerance acquisition enhances BV-2 microglial migration and phagocytosis via ERK1/2 signaling, but these effects are attenuated under hypoxic conditions. These findings provide new insights into hypoxia-driven modulation of tolerant microglia with potential implications for neuroinflammation and ischemic brain injury.

**Abstract:**

Background: Prolonged hypoxia contributes to irreversible organ damage, particularly in the brain and heart. While chronic hypoxia is harmful, mild short-term hypoxia can trigger protective mechanisms. This study investigates how such hypoxic conditions affect BV-2 tolerant microglial cells in vitro, focusing on inflammation, metabolism, and functional activity. Although in vitro models provide a controlled setting, our findings may offer insights into microglial behavior in vivo under similar conditions. Methods: We used various molecular and biochemical techniques to assess the inflammatory state of BV-2 microglia under hypoxia, measuring glycolytic activity (via lactate production), and evaluating migratory and phagocytic capacities in vitro. Results: Hypoxic conditions induced a more tolerant, anti-inflammatory phenotype in BV-2 cells, with decreased pro-inflammatory mediators and reduced glycolytic activity, regulated by the MyD88/NF-κB p65 pathway. Tolerance supports increased migration and phagocytosis, but under hypoxic conditions, these effects were significantly declined compared to normoxic conditions, mediated through the ERK1/2 pathway. Conclusions: These findings suggest that short-term hypoxia may regulate microglial behavior and restore homeostasis, with implications for neuroinflammatory conditions.

## 1. Introduction

Microglial cells, the resident self-renewing innate immune cells of the central nervous system (CNS), are essential for immune surveillance and neurodevelopment [1,2,3]. They act as immune sentinels controlling the patterning and wiring of the brain in early development, clearing pathogens and contribute to homeostasis throughout life [4,5,6].

Microglia play a critical role in neuronal maturation, synapse formation and pruning, and homeostatic regulation during the neonatal period by engaging in phagocytosis, antigen presentation, and the regulation of neuroinflammation [7,8]. They primarily originate from the yolk sac and migrate to the developing brain during embryogenesis, where they initiate surveillance and neuroinflammatory activities, although their full maturation occurs postnatally [9,10,11,12]. A variety of parenchymal events such as the release of damage-associated molecular pattern molecules (DAMPs) from impaired tissue or insults, as well as infectious events mediated by small molecular motifs termed pathogen molecular pattern molecules (PAMPs), trigger microglial activations accompanied by increased production of inflammatory cytokines, generation of reactive oxygen species (ROS), increased motility and phagocytic activities [2,3,5,13]. Furthermore, recent reports demonstrated that early glycolytic reprogramming represent a major metabolic regulator of microglial activation, enabling microglia faster adenosine triphosphate (ATP) production resulting in the generation of cell growth intermediates and cytokine release [14,15]. While microglial activation is essential for an effective immune response, dysregulation of microglial activity can lead to pathological consequences, exacerbating tissue damage and contributing to the onset of neurodegenerative disorders, highlighting their dual role in both CNS immunity and neurodevelopment [10,16,17]. Maintaining a balance in microglial activity, between driving inflammatory responses to support protective functions and limiting excessive inflammation to prevent tissue damage, is crucial for the proper development and homeostasis of the CNS.

Lipopolysaccharide (LPS), a potent endotoxin, is recognized by toll-like receptor 4 (TLR4), initiating a cascade of molecular events that culminate in the translocation of nuclear factor kappa B (NF-κB) to the nucleus, driving the production of a range of inflammatory mediators [18,19]. Prolonged exposure to LPS induces a temporary innate memory unresponsive state characterized by a decreased release of pro-inflammatory elements known as endotoxin tolerance [20,21]. Induction of immune tolerance may increase susceptibility to secondary infections, but it can also be beneficial by limiting excessive inflammatory responses, thereby promoting repair mechanisms. This balance is particularly crucial for successful fetal development and pregnancy [22,23,24]. Fetomaternal immune tolerance is not limited to T lymphocytes, but is associated largely with innate immune cells [25,26,27]. Having into consideration the fact that microglia colonizes the CNS during embryotic development, maintaining immune tolerance reactions during this phase is critical to avoid any possible neuroinflammatory disorders or neurodevelopmental defects [28,29,30].

The brain represents one of the most oxygen- and energy-demanding organs, making it particularly susceptible to oxygen deprivation, which can lead to hypoxic injury and, if prolonged, potentially cause irreversible neuronal damage [31,32]. Fetal life evolves in a hypoxic environment, with the oxygen partial pressure (PaO_2_) being lower than that in postnatal life due to the placental barrier [33,34,35,36]. Known as physiological fetal hypoxia, it usually affects the fetal development from the 23rd–36th week of gestation, subjected initially with slow and progressive reduction of oxygen supply through the placenta [37,38]. However, the impact of physiological fetal hypoxia to the fetomaternal immune-tolerant microglia remains elusive.

In this study, we have elaborated the role of hypoxia in immune-tolerant microglia in vitro. To this end, we developed a two-step protocol to induce tolerance reactions in BV-2 microglial cells under both normoxic and hypoxic conditions. Our data demonstrated that hypoxia fosters attenuated inflammatory responses and enhanced tolerance reactions in microglia compared to normoxic conditions, mediated by the myeloid differentiation primary response gene 88 (MyD88)–NFκB-p65 pathway. Metabolically, these effects were linked by reduced glycolysis characterized by declined levels of lactate production. Functionally, microglial motility and phagocytic activity were significantly increased in the tolerant microglia, with marked differences observed between normoxic and hypoxic conditions, primarily mediated by the extracellular signal-regulated kinase (ERK) 1/2 pathway.

## 2. Materials and Methods

### 2.1. BV-2 Cell Culture

The immortalized murine microglial BV-2 cell line was kindly provided by Prof. Dr. Reinhard Bauer research laboratory, Institute for Molecular Cell Biology, Jena, Germany [39,40,41]. The suitability of BV-2 microglial cells compared to other available murine microglial cell lines for our study was determined based on several key criteria: (i) they serve as a relevant in vitro model to study neonatal neuroinflammation; (ii) they retain essential microglial functions such as cytokine production, migration, and efficient phagocytosis; (iii) they offer a standardized, easily cultured alternative with consistent phenotypic and functional properties; and (iv) they are well-characterized with defined signaling pathways involved in various inflammatory responses, retaining similar signaling pathways in neuroinflammation as activated neonatal microglia.

Cells were cultured in T75 flasks using Dulbecco’s Modified Eagle Medium (DMEM; Cat. No. D6429, Sigma-Aldrich, St. Louis, MO, USA) supplemented with 10% heat-inactivated fetal calf serum (hiFCS; Cat. No. PB-FCS-EU-0500, PeloBiotech, Planegg, Germany), 1% penicillin/streptomycin (Cat. No. P4333, Sigma-Aldrich), and 1% amphotericin B (Cat. No. A2942, Sigma-Aldrich). Cultures were maintained at 37 °C in a humidified atmosphere containing 5% CO_2_. Upon reaching the desired confluence, cells were detached using a sterile solution of 2 mM ethylenediaminetetraacetic acid (EDTA) in Dulbecco’s phosphate-buffered saline (DPBS; Cat. No. D8537, Sigma-Aldrich) and then seeded onto 6-well plates for subsequent experiments.

### 2.2. Stimulation Procedure and Hypoxia Induction in Vitro

**Figure 1:**
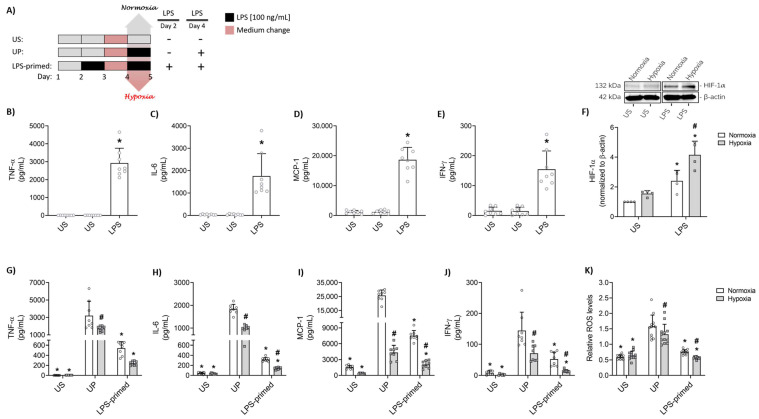
Cytokine, chemokine, and ROS response to LPS stimulation and LPS-priming under normoxic and hypoxic conditions in BV-2 microglia in vitro. (**A**) Schematic illustration of the two-step protocol used to induce endotoxin tolerance and study its effects under normoxic and hypoxic conditions. BV-2 microglial cells (100,000 cells/well) were stimulated by a fixed dose of LPS (100 ng/mL) on day 1 for 24 h and supernatants were analyzed for their inflammatory cytokine and chemokine production of: (**B**) TNF-α, (**C**) IL-6, (**D**) MCP-1, (**E**) IFN-γ (*N* = 8) analyzed by ELISA. Protein lysates 24 h after the stimulation under normoxic or hypoxic conditions were analyzed for their (**F**) HIF-1α (*N* = 4) protein expression by Western blotting and quantified (unstimulated cells under normoxia assigned as 1.0). The medium was changed (stimuli washed-out) and after the resting time, cells were challenged by LPS (100 ng/mL) on day 4 under normoxic and hypoxic conditions for 24 h. Supernatants have been collected after the second challenge and analyzed for their levels of: (**G**) TNF-α, (**H**) IL-6, (**I**) MCP-1, (**J**) IFN-γ (*N* = 8) assessed by ELISA. (**K**) ROS levels (*N* = 4, with *n* = 3 replicates per experiment) after the second challenge were measured by DCFDA assay. Results are depicted as scatter dot plots with mean + SD. For panels A-E, statistical comparisons were made using a 1-way ANOVA (parametric) or Kruskal–Wallis test (non-parametric), as appropriate. For panels F-K, a 2-Way ANOVA was used. Significance is indicated by * *p* < 0.05, * versus unstimulated (US, **B**–**F**) or unprimed (UP, **G**–**K**) control; # *p* < 0.05, # versus normoxic condition (white bar).

BV-2 microglial cells (1 × 10^5^ cells/well) were stimulated according to the well-established two-step protocol, as depicted using DMEM medium supplemented with 2% hiFCS (Figure 1A). Briefly, cells were first pre-stimulated with a fixed dose of LPS (100 ng/mL; *E. coli* 055:B5; Cat. No. tlrl-pb5lps, InvivoGen, Toulouse, France) on day 2 for 24 h at 37 °C and 5% CO_2_. After this, the supernatants were collected, the medium was replaced, and the cells were allowed to rest overnight (37 °C, 5% CO_2_). On day 4, cells were then re-challenged with LPS (100 ng/mL) and incubated at 37 °C and 5% CO_2_ under either normoxic (20% O_2_) or hypoxic conditions. Hypoxia was induced using a BD GasPak EZ Container (Becton Dickinson, Franklin Lakes, NJ, USA) packed with Oxoid™ sachets, generating a hypoxic environment of <1% O_2_. RNA samples were collected 6 h after the second LPS challenge, while supernatants and protein lysates were collected 24 h post-stimulation with LPS. Both controls, the unstimulated (US; cultivated only in medium) and unprimed (UP; stimulated once on day 4 with 100 ng/mL LPS) conditions, were included for cells under both normoxia and hypoxia groups.

### 2.3. Enzyme-Linked Immunosorbent Assay (ELISA)

Cell culture supernatants from BV-2 cells, collected after the first challenge as well as those exposed to normoxic and hypoxic conditions 24 h following the second LPS challenge, were analyzed for inflammatory cytokines and chemokines. Commercial ELISA kits for TNF-α (Cat. No. 430901), IL-6 (Cat. No. 431301), IFN-γ (Cat. No. 430801) and MCP-1 (Cat. No. 432701) were obtained from BioLegend (San Diego, CA, USA) [42,43]. Briefly, the supernatants were prepared and applied to a 96-well plate pre-coated with the primary capture antibody, incubated for 2 h at room temperature. Following a washing step (PBS + 0.05% Tween-20), the detection antibody was added and incubated for 1 h at room temperature. The plate was washed three times, and 100 µL/well of diluted Avidin-HRP solution was introduced. After a 30 min incubation, the plate was washed again, and 100 µL/well of BD OptEIA™ TMB Substrate Reagent (Cat. No. 555214, Becton Dickinson) was added. The plate was incubated for 15–30 min, allowing color development to reach the desired intensity. The reaction was then stopped, and the absorbance was measured at 450 nm, with a reference wavelength of 570 nm, using an iMark microplate reader (Bio-Rad Laboratories, Hercules, CA, USA). Cytokine and chemokine concentrations were determined based on the absorbance values obtained from the standard curve.

### 2.4. Antibodies

Antibodies for Sodium Dodecyl Sulfate-Polyacrylamide Gel Electrophoresis (SDS-PAGE) Western Blotting were sourced from Cell Signaling (Danvers, MA, USA): MyD88 (Cat. No. 4283), p44/42 MAPK (ERK1/2) (Cat. No. 9107), phospho-p44/42 MAPK (ERK1/2) (Thr202/Tyr204) (Cat. No. 9106), and phospho-NF-κB p65 (Cat. No. 3033). The primary antibody for HIF-1α (Cat. No. sc-13515) was obtained from Santa Cruz Biotechnology, Inc. (Dallas, TX, USA). β-actin (Cat. No. A5441, Sigma-Aldrich) was used as a loading control. Horseradish peroxidase (HRP)-conjugated secondary antibodies, anti-rabbit (Cat. No. 111-035-144) and anti-mouse (Cat. No. 115-035-166), were purchased from Dianova (Hamburg, Germany).

### 2.5. Protein Extraction and SDS-PAGE Western Blotting

BV-2 microglial cells were lysed in 200 μL radioimmunoprecipitation assay (RIPA) buffer supplemented with freshly prepared Pierce Protease and Phosphatase Inhibitor tablets (Cat. No. A32959, Thermo Fisher Scientific, Waltham, MA, USA) as per standard protocols [44,45]. The lysates were vortexed and then immediately centrifuged at 12,000× *g* for 30 min at 4 °C to remove cellular debris. The resulting supernatants were collected and their protein concentrations were determined using the Bradford assay, with commercially available kits (Pierce 660 nm; Cat. No. 22662, Thermo Fisher Scientific). For Western blot analysis, 40 µg of total protein was mixed with sample buffer (5% SDS, 33% glycerol, 25% β-mercaptoethanol) and heated at 95 °C for 5 min to denature the proteins. The samples were then subjected to SDS-PAGE on a 10% polyacrylamide gel, followed by transfer to a polyvinylidene fluoride (PVDF) membrane. The membrane was blocked with 1% bovine serum albumin (BSA) for 30–45 min, then incubated overnight at 4 °C with primary antibodies. After incubation, the membrane was washed three times with Tris-buffered saline containing 0.1% Tween-20 (TBS-T), then incubated with HRP-conjugated secondary antibodies for 1 h at room temperature. Following another round of washing, protein bands were visualized using enhanced chemiluminescence (ECL) reagents and imaged with the Chemi-Doc XRS+ System (Bio-Rad Laboratories, Hercules, CA, USA). Protein band intensities were quantified using Image Lab Ver. 6.0.1 software (Bio-Rad Laboratories).

### 2.6. Measurement of Reactive Oxygen Species (ROS)

The production of reactive oxygen species (ROS) in BV-2 cells was assessed using the DCFDA Cellular ROS Assay Kit (Cat. No. ab113851, Abcam, Cambridge, UK), in accordance with the manufacturer’s instructions. Briefly, BV-2 microglia (5000 cells/well) were stimulated in a black, clear-bottom 96-well plate as described in the protocol, and were maintained under either normoxic or hypoxic conditions as previously outlined. Prior to the second LPS challenge, the DMEM complete medium was removed, and the cells were washed with 1× Assay Buffer (100 µL/well). The cells were then incubated with 100 µL/well of a 20 μM 2′,7′-dichlorofluorescin diacetate (DCFDA) solution under dark conditions for 45 min at 37 °C. After incubation, the DCFDA solution was removed, and cells were washed with 1× Assay Buffer (100 µL/well). Following this, the cells were then treated with the second challenge of LPS (100 ng/mL) for 6 h. DCFDA enters live cells, where it is deacetylated by esterases into a non-fluorescent intermediate, which is then converted into the fluorescent 2′,7′-dichlorofluorescein (DCF) upon oxidation by ROS. The resulting fluorescence (excitation/emission: 485 nm/535 nm) was measured using a Fluoroskan™ FL plate reader (Thermo Fisher Scientific).

### 2.7. Lactate Measurements

Lactate production in cell culture supernatants was quantified using the L-Lactate Assay Kit (Cat. No. 8308, ScienCell Research Laboratories, Carlsbad, CA, USA) according to the manufacturer’s protocol [46]. A 4 mM L-Lactate standard solution was prepared by adding 50 µL of L-Lactate standard to 200 µL of Assay Buffer. To generate a serial dilution, 200 µL of Assay Buffer was added to seven labeled test tubes. A 200 µL aliquot of the 4 mM L-Lactate solution was transferred to the first tube and mixed, yielding a 2 mM standard. This process was repeated sequentially to create a dilution series, halving the lactate concentration in each subsequent tube, with the seventh tube serving as the blank containing only Assay Buffer. About 50 µL of each standard, test sample, and blank were aliquoted in duplicate into a 96-well plate.

For lactate measurement, 2 µL of reconstituted enzyme mix was added to each well containing standards, test samples, or blanks. Subsequently, 50 µL of Substrate Mix was added to each well, and the plate was incubated for 20 min at room temperature in the dark. Absorbance was measured at 490 nm using an iMark microplate reader (Bio-Rad Laboratories) to quantify lactate levels in the samples.

### 2.8. In Vitro Transmigration Assay

The migratory capacity of BV-2 microglial cells was assessed using the transwell migration assay, as previously described [47]. A 100 μL cell suspension, containing 1 × 10^5^ cells in 0.5% FCS medium, was carefully pipetted onto the membrane of a 24-well transwell insert (8 μm pore size) and incubated for 10 min at 37 °C in a 5% CO_2_ atmosphere. Following this, 600 μL of migration buffer, containing 20% hiFCS as a chemoattractant, was added to the well below the insert. The plate was incubated for 4 h at 37 °C and 5% CO_2_ to allow cell migration. After the incubation period, any non-migrated cells on the apical side of the insert membrane were gently removed using a cotton-tipped applicator. The migrated cells on the basal side were fixed by immersing the transwell insert in 1 mL of 4% sterile-filtered paraformaldehyde (PFA) for 10–15 min at room temperature. The inserts were then removed and allowed to dry before proceeding with 4′,6-diamidino-2-phenylindole (DAPI) staining.

Fluorescence staining was performed by adding 600 μL of DAPI (1 μg/mL) to the 24-well plate containing the transwell inserts and incubated for 10 min, protected from light. After incubation, the inserts were washed thrice with PBS to remove excess dye. The inserts were then allowed to dry and mounted onto microscope slides (Epredia X50 SuperFrost slide 26 × 76 mm, Cat. No. AA00008032E01MNZ10, Thermo Fisher Scientific). Images of the migrated cells on the transwell membrane were captured using a Leica Microsystems Ltd. fluorescence microscope (DFC450-Camera, version 4.9.0.; Leica Microsystems, Wetzlar, Germany) with a 10× objective to visualize the cells. The number of migrated cells (DAPI-stained nuclei) was counted in five independent visual fields and expressed as the absolute number of transmigrated cells.

### 2.9. In Vitro Phagocytosis Assay

The phagocytic activity of BV-2 microglia was evaluated using the CytoSelect™ 96-well Phagocytosis Assay (Cell Biolabs, Cat. No. CBA-224, San Diego, CA, USA), following the manufacturer’s protocol. Briefly, BV-2 microglial cells (5 × 10^4^ cells/well) were seeded in 96-well plates and exposed to either normoxic or hypoxic conditions as stated above. A 10 µL suspension of Zymosan (*Saccharomyces cerevisiae*) particles was added to each well and incubated for 2 h at 37 °C in a 5% CO_2_ atmosphere. Following incubation, the cells were subjected to multiple washing steps and subsequently fixed according to the manufacturer’s instructions. The plate was then incubated with blocking buffer, followed by a brief permeabilization step and additional washes with PBS. Internalized Zymosan particles were quantified by measuring the absorbance at 405 nm using an iMark microplate reader (Bio-Rad Laboratories). Phagocytic activity was expressed as a percentage of the absorbance values of the unstimulated control under normoxic conditions, which was set as 100%.

### 2.10. Analysis of Cell Viability and Cytotoxicity

Cell viability and cytotoxicity of BV-2 microglia under normoxic and hypoxic conditions were assessed as previously described [44,45]. For both viability and cytotoxicity assays, 5000 cells/well were seeded and treated as outlined above. 24 h after the second LPS challenge, 10 µL of MTT labeling reagent (5 mg/mL) was added to each well, followed by a 4 h incubation at 37 °C. After incubation, 100 µL of solubilization solution (0.01 M HCl and 20% SDS) was added, and the plate was incubated overnight. Cytotoxicity was evaluated using a colorimetric Cell Cytotoxicity Assay Kit (Cat. No. ab112118, Abcam). 20 µL of assay solution was added to each well, and the plate was incubated at 37 °C with 5% CO_2_ for 4 h, protected from light.

Absorbance was measured at 570 nm using an iMark microplate reader, with data presented as relative viability or cytotoxicity, where the unstimulated control was set to 100%.

### 2.11. RNA Isolation and Real-Time qPCR

To assess gene expression levels, total RNA was extracted from BV-2 microglial cells 6 h after the second LPS challenge under normoxic or hypoxic conditions using TRIzol™ Reagent (Cat. No. 15596018, Thermo Fisher Scientific). RNA concentration and quality were evaluated with a Nanodrop DS11 FX+ spectrophotometer (DeNovix, Wilmington, NC, USA). Complementary DNA (cDNA) was synthesized using the High-Capacity cDNA Reverse Transcription Kit (#4368814, Applied Biosystems, Waltham, MA, USA). Real-time quantitative PCR (qPCR) was carried out on a StepOnePlus system (Applied Biosystems). The primers used in this study are listed in Table 1.

GAPDH was used as the housekeeping gene, and relative gene expression was calculated using the comparative C_T_ (2^−ΔΔC^_T_) method [48].

### 2.12. Statistical Analysis

Data are presented as means + standard deviation (SD), and statistical analyses were conducted using SigmaPlot Software version 12.0 (Systat Software GmbH, Erkrath, Germany). Graphs were generated with GraphPad Prism Software version 8.0.2 (GraphPad Software, San Diego, CA, USA). Between-group comparisons were performed using one-way or two-way analysis of variance (ANOVA), as appropriate. If normality test failed, the non-parametric Kruskal–Wallis on ranks was applied. Post hoc analyses were carried out using either the Holm–Sidak test or Dunn’s method, depending on the context. A *p*-value of <0.05 was considered statistically significant.

## 3. Results

### 3.1. Hypoxia Potentiates LPS-Mediated Tolerance in BV-2 Microglial Cells

Initial stimulation of BV-2 microglial cells with LPS (100 ng/mL) for 24 h led to a pronounced increase in the secretion of key inflammatory mediators compared to the unstimulated counterparts. ELISA measurements showed significantly elevated concentrations of TNF-α, IL-6, and IFN-γ, alongside increased production of the chemokine monocyte chemoattractant protein-1 (MCP-1) (Figure 1B–E).

Subsequently, the expression of hypoxia-inducible factor (HIF)-1α was assessed by Western blotting to investigate the effects of oxygen tension on BV-2 microglial cells in vitro. LPS stimulation enhanced HIF-1α expression in BV-2 cells under both normoxic and hypoxic conditions. Notably, while hypoxia alone tends to increase the HIF-1α levels compared to normoxia, the combination of hypoxia and LPS stimulation led to a further significant upregulation of HIF-1α compared to LPS treatment under normoxic conditions (Figure 1F).

To explore microglial adaptive responses, we established a two-step protocol designed to induce endotoxin tolerance in BV-2 microglia as described above. Using this approach, the effect of hypoxia on tolerance development following LPS priming was examined. Unstimulated (US) BV-2 microglia expressed low basal levels of inflammatory cytokines and chemokines (Figure 1G–J, *p* < 0.05). A single challenge with 100 ng/mL LPS (unprimed, UP) led to a marked increase in levels of TNF-α, IL-6, MCP-1 and IFN-γ under both normoxia and hypoxia counterparts (*p* < 0.05). When comparing LPS-primed cells to the UP state, tolerance induction was evident. Notably, hypoxic conditions were associated with a more pronounced reduction in the production of these pro-inflammatory cytokines and chemokines in LPS-primed cells relative to normoxia (Figure 1G–J). The inclusion of two ‘US’ conditions in Figure 1B–E accurately reflects the experimental timeline, where the first ‘US’ represents unstimulated cells, and the second ‘US’ remains unchanged until day 4. On day 4, the second ‘US’ group is stimulated with LPS and transitions to the ‘UP’ (unprimed) condition, as depicted in the schematic illustration.

Prior to analyzing the inflammatory responses, we evaluated cell viability and cytotoxicity under the selected LPS concentration and hypoxic conditions to exclude treatment-related cell damage in vitro (Supplementary Materials Figure S1).

Moreover, ROS production was also evaluated and showed a consistent reduction under hypoxic conditions in tolerant cells, paralleling the cytokine expression pattern (Figure 1K).

### 3.2. Glycolysis Mediates LPS-Induced Tolerance in BV-2 Microglia

**Figure 2:**
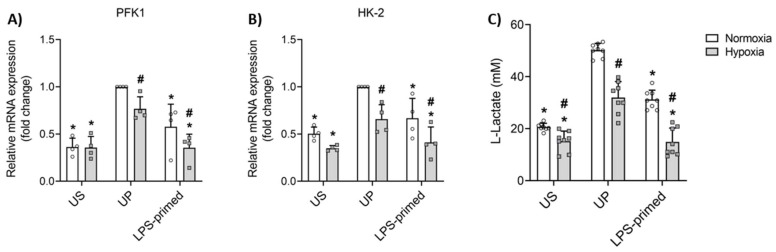
Glycolytic metabolism mediates LPS-priming effects in BV-2 microglia under normoxic and hypoxic conditions in vitro. BV-2 cells after the second challenge on day 4 (100 ng/mL LPS) under normoxic and hypoxic conditions were analyzed for gene expression of (**A**) phosphofructokinase 1 (PFK1), and (**B**) hexokinase 2 (HK-2) (*N* = 4, normalized to GAPDH; unprimed cells under normoxia assigned as 1.0) by real-time PCR. (**C**) Lactate production (*N* = 8) in cell culture supernatants was measured using the commercially available L-Lactate Assay Kit. Results are depicted as scatter dot plots, mean + SD, 2-Way ANOVA, * *p* < 0.05, * versus unprimed (UP) control; # *p* < 0.05, # versus normoxic condition (white bar).

In addition to the inflammatory responses, we further investigated the impact of hypoxia on the glycolytic metabolism of LPS-primed tolerant BV-2 microglial cells in vitro. Hypoxia has been implicated in regulating metabolic pathways, particularly influencing glycolysis across various cell types. Our data indicate that LPS-primed tolerant BV-2 cells exhibit a significant reduction in the expression of key glycolytic enzymes, including phosphofructokinase (PFK) 1 and hexokinase (HK)-2, under both normoxic and hypoxic conditions (Figure 2A,B).

Moreover, the production of L-lactate, a byproduct of glycolysis, was markedly decreased in tolerant cells (Figure 2C), consistent with the reduced expression of glycolytic genes. Interestingly, hypoxia appeared to induce a more pronounced reduction in both glycolytic gene expression and lactate levels compared to normoxic conditions, highlighting the unique metabolic response of BV-2 cells under hypoxic stress.

### 3.3. MyD88-Dependent Suppression of NFκB-p65 Supports LPS-Induced Tolerance Under Normoxic and Hypoxic Conditions

Besides the inflammatory responses and glycolytic alterations observed, we further investigated the underlying signaling pathways involved in the modulation of inflammation in BV-2 microglial cells in vitro. Specifically, we analyzed the role of MyD88, an adaptor protein involved in TLR4-mediated signaling, and NF-κB p65 (RelA), a critical transcription factor driving pro-inflammatory gene expression.

**Figure 3:**
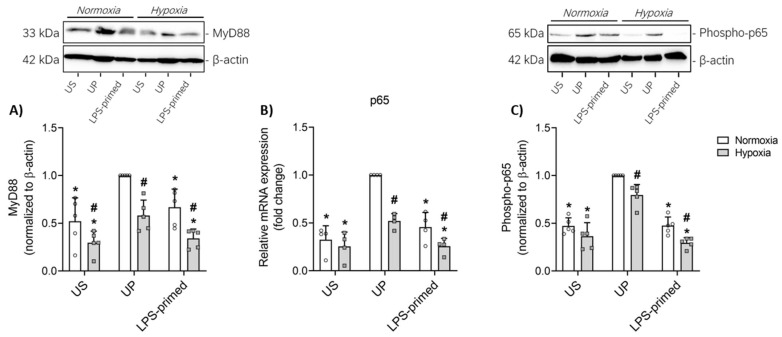
MyD88-dependent regulation of NFκB-p65 contributes to LPS-induced tolerance in vitro. BV-2 cells after the second challenge on day 4 (100 ng/mL LPS) under normoxic and hypoxic conditions were analyzed for (**A**) MyD88 (*N* = 5) and (**C**) phospho-p65 (*N* = 5) protein expression measured by Western blotting and quantified (unprimed cells under normoxia assigned as 1.0). Gene expression of (**B**) NFκB-p65 (*N* = 4, normalized to GAPDH; unprimed cells under normoxia assigned as 1.0) was assessed by real-time PCR. Results are depicted as scatter dot plots, mean + SD, 2-Way ANOVA, * *p* < 0.05, * versus unprimed (UP) control; # *p* < 0.05, # versus normoxic condition (white bar).

Under unprimed conditions, both MyD88 and phospho-p65 protein levels as well as p65 gene expression were significantly elevated in BV-2 microglia compared to UP controls (Figure 3A–C). In LPS-primed BV-2 cells, which exhibited a tolerance phenotype with reduced inflammatory output, we observed a marked reduction in both MyD88 and phospho-p65 protein levels as well as p65 gene expression.

Consistent with our inflammatory and glycolytic findings, the expression of both MyD88 and p65 was significantly reduced under hypoxic conditions compared to normoxic counterparts (Figure 3).

### 3.4. LPS-Induced Tolerance Promotes Increased Migration of BV-2 Microglia Mediated by ERK1-2 Pathway

**Figure 4:**
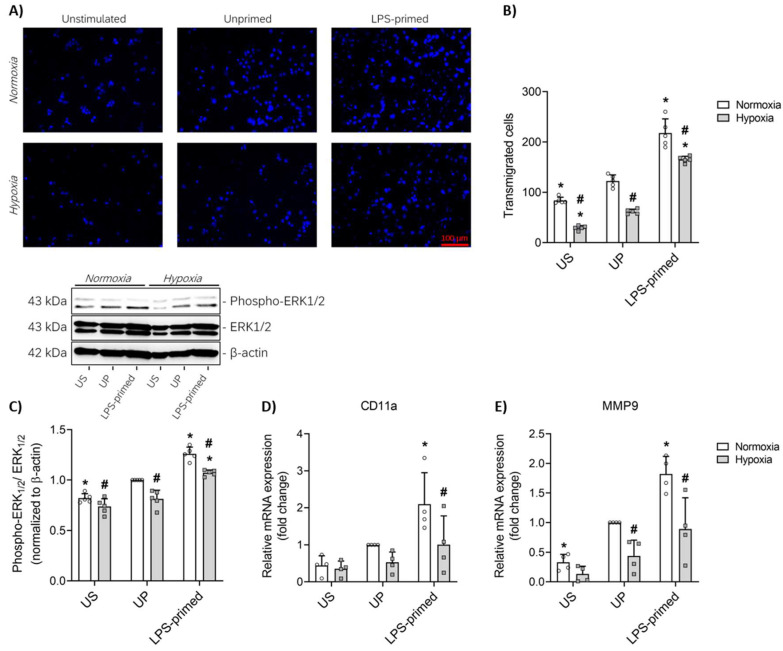
LPS-primed BV-2 microglia exhibit enhanced migratory activity mediated by ERK1/2 signaling pathway. BV-2 cells after the second challenge on day 4 (100 ng/mL LPS) under normoxic and hypoxic conditions were analyzed for their (**A**) migratory ability using the in vitro transwell migration assay and (**B**, *N* = 5) the number of migrated cells (DAPI-stained nuclei) was expressed as the absolute number of transmigrated cells. Protein expression of (**C**) phospho-ERK1/2 (*N* = 5) were assayed by Western blotting and quantified (unprimed cells under normoxia assigned as 1.0). RNA samples collected 6 h after the second challenge were analyzed for gene expression of (**D**) CD11a and (**E**) MMP9 (*N* = 4, normalized to GAPDH; unprimed cells under normoxia assigned as 1.0) by real-time PCR. Results are depicted as scatter dot plots, mean + SD, 2-Way ANOVA, * *p* < 0.05, * versus unprimed (UP) control; # *p* < 0.05, # versus normoxic condition (white bar).

Beyond the previously characterized inflammatory and metabolic alterations, we next assessed the migratory behavior of BV-2 microglial cells—a crucial functional aspect underlying their neuroprotective and surveillance roles. Using a transwell migration assay, we observed that tolerized BV-2 cells (LPS-primed) exhibited a significantly enhanced migratory capacity compared to unprimed controls (*p* < 0.05, Figure 4A,B), indicating a functional adaptation linked to their prior inflammatory conditioning. In contrast, BV-2 cells subjected to hypoxic conditions demonstrated a marked reduction in migratory activity relative to cells maintained under normoxic conditions (Figure 4A,B).

To further dissect the molecular basis of these migratory differences, we assessed the expression of two key markers implicated in microglial motility: integrin subunit αL (CD11a) and matrix metallopeptidase (MMP) 9. CD11a, an integrin subunit involved in adhesion and transendothelial migration, was significantly upregulated in LPS-tolerized cells, whereas hypoxic cells displayed a reduced expression profile (Figure 4D). Similarly, MMP9, which facilitates extracellular matrix remodeling during cell movement, was elevated in the LPS-primed condition but markedly diminished under hypoxia (Figure 4E).

Mechanistic analysis using Western blotting revealed that the enhanced migration observed in tolerized BV-2 microglial cells was accompanied by increased phosphorylation of ERK 1/2 (Figure 4C), indicating that the mitogen-activated protein kinase (MAPK)/ERK signaling pathway plays a supportive role in mediating the pro-migratory activity of these cells.

### 3.5. LPS-Induced Tolerance Triggers Increased Phagocytic Activity Under Normoxic and Hypoxic Conditions

In addition to migration, we next examined phagocytosis as a critical function of microglial cell clearance, essential for maintaining CNS homeostasis and immune resolution. Using a commercially available fluorescent-based phagocytosis assay, we quantified the capacity of BV-2 microglia to internalize zymosan particles under different experimental conditions in vitro.

**Figure 5:**
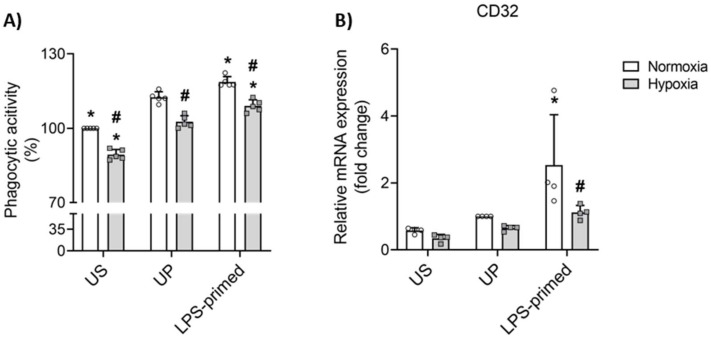
LPS-primed BV-2 microglia triggers increased phagocytic activity in BV-2 microglia in vitro. BV-2 cells after the second challenge on day 4 (100 ng/mL LPS) under normoxic and hypoxic conditions were analyzed for their (**A**, *N* = 5) phagocytic activity using a CytoSelect 96-Well Phagocytosis Assay kit and quantified (unstimulated cells under normoxia assigned as 100%). RNA samples collected 6 h after the second challenge were analyzed for gene expression of (**B**) CD32 (*N* = 4, normalized to GAPDH; unprimed cells under normoxia assigned as 1.0) by real-time PCR. Results are depicted as scatter dot plots, mean + SD, 2-Way ANOVA, * *p* < 0.05, * versus unprimed (UP) control; # *p* < 0.05, # versus normoxic condition (white bar).

LPS-primed BV-2 microglial cells demonstrated a significantly enhanced phagocytic activity compared to UP cells (*p* < 0.05), whereas cells cultured under hypoxic conditions displayed a significant reduction in phagocytic capacity when compared to those maintained under normoxic conditions in vitro (Figure 5A).

We further analyzed the expression of CD32, a low-affinity Fc receptor that facilitates the recognition and internalization of opsonized particles, playing a central role in immune complex clearance responses. Gene expression analyses revealed that CD32 expression was significantly upregulated in LPS-primed BV-2 cells compared to UP control, consistent with their increased phagocytic function (Figure 5B). Conversely, CD32 levels were markedly decreased under hypoxic conditions compared to normoxic counterparts (Figure 5B).

## 4. Discussion

Microglial cells, known trivially as macrophage-like cells of CNS, represent surveillance sensors in charge of maintaining and safeguarding brain homeostasis [1,2,8]. Ontogenetically, microglial cells begin their development early, around 4.5 weeks of gestation during the first trimester, alongside other innate immune cells, and subsequently migrate to colonize the brain [10,49]. In our study, BV-2 cells were selected due to several compelling reasons, as they share numerous characteristics with primary microglial cells, such as: (i) similar response patterns to PAMPs, particularly LPS, which activates NF-κB signaling; (ii) comparable functional responses, such as the production of cytokines and chemokines relevant to neuroinflammation; (iii) retention of key signaling pathways involved in inflammatory responses, mirroring those of activated neonatal microglia; (iv) transcriptomic profiles that partially resemble those of primary microglia; (v) the capacity for cell-to-cell interactions that can activate other glial cells (e.g., astrocytes); (vi) overlapping metabolic responses, especially for glycolytic activity; and (vii) comparable functional properties, including migration and phagocytosis [50,51,52,53,54,55].

Surface pattern-recognition receptors (PRRs) represent a critical feature of innate immune cells in general, including microglia as the main innate immune cell in CNS, for recognizing various conserved pathogenic (e.g., PAMPs) or endogenous (e.g., DAMPs) structures [56,57]. Activation of PRRs results in a variety of downstream signaling events causing the NF-κB activation and triggering the release of a variety of inflammatory mediators, including cytokines, chemokines as well as the production of ROS [58,59]. LPS as a potent PAMP derived from Gram-negative bacteria is well known agonist of TLR4, triggering the release of a variety of pro-inflammatory cytokines (i.e., TNF-α, IL-6, IFN-γ) and chemokines (i.e., MCP-1) [18,19]. Our results demonstrated that in vitro stimulation of BV-2 microglial cells with LPS triggered increased production of TNF-α, IL-6, IFN-γ, and MCP-1. In line with this, several studies have shown that LPS stimulation triggers an extraordinarily potent inflammatory reaction with increased levels of inflammatory cytokines and chemokines via NF-κB [13,60,61]. These findings, particularly the elevated levels of TNF-α, IL-6, MCP-1 and IFN-γ, indicate that BV-2 cells mount a potent inflammatory response, akin to that of primary microglia, thereby contributing to the neuroinflammatory milieu. Furthermore, it is well established that under hypoxic conditions, HIF-1α protein escapes proteasomal degradation, leading to its stabilization [62,63]. As a key regulator of cellular responses to low oxygen levels, HIF-1α is continuously synthesized and degraded under normal conditions, exhibiting a very short half-life [62,64]. However, under low oxygen (hypoxic) tension, the degradation of HIF-1α is significantly suppressed, allowing its accumulation and activation [65,66]. Consistent with these findings, our data show that BV-2 microglial cells under hypoxic conditions exhibit increased levels of HIF-1α protein, with a particularly marked increase following LPS stimulation in vitro. This observation suggests that, similar to primary microglia, hypoxic conditions can induce HIF-1α stabilization in BV-2 cells, further augmented by LPS-induced inflammatory signaling. This difference, compared to the basal state, can be attributed to LPS-induced inflammation, which increases cellular activity and metabolic demand, leading to localized oxygen depletion and microregional hypoxia despite an overall normoxic environment [67,68].

Development of an appropriate inflammatory response is the key element to afford proper resistance reactions against invading pathogens; however, uncontrolled induction of pro-inflammatory mediators may contribute to the progression of host tissue injury. Fetomaternal tolerance is crucial for the maintenance of a healthy pregnancy, as it ensures that the maternal immune system does not mount an aggressive response against the developing fetus, which is genetically distinct [22,23,25]. As a non-specific phenomenon, tolerance, is not limited to any specific receptor or signaling element, and can be induced by a variety of stressing factors [21,69]. Nonetheless, LPS-induced tolerance, however, is one of the most well-characterized and earliest known forms of immune adaptation [70,71,72]. Physiologically, LPS is a large, polar molecule that ordinarily has limited ability to cross the placental barrier. However, under inflammatory conditions, the integrity and permeability of the placenta are compromised, allowing LPS directly or indirect by its downstream inflammatory mediators (e.g., cytokines) to gain access to the fetal compartment, resulting in significant implications for the fetus [73,74,75]. As observed in our study, immune tolerance in BV-2 microglia develops following repeated exposure to high doses of LPS, characterized by a reduced expression of pro-inflammatory cytokines and oxidative stress mediated by ROS. Interestingly, BV-2 cells under hypoxic conditions exhibited a stronger tolerant reaction compared to cells under normoxia. These findings are consistent with recent studies showing that short-term preconditioning with moderate hypoxia enhances tolerance in LPS-induced endotoxemia [76,77]. These effects might be driven by corticosterone, which promotes adenosine release and stimulates adenosine A2B receptors, thereby suppressing excessive inflammatory responses [77,78]. Mechanistically, LPS-induced tolerance in BV-2 microglia under both normoxic and hypoxic conditions is driven by the interaction between MyD88, the adaptor protein for TLR4 signaling, and the NF-κB p65 transcription factor. LPS activates the canonical NF-κB pathway through TLR/MyD88 signaling, initiating downstream events that involve the degradation of IκBα by the IκB kinase (IKK) complex [58,79]. This degradation leads to the rapid translocation of NF-κB to the nucleus, where it regulates the production of inflammatory mediators [58,79,80]. Similarly, our group’s data, along with other studies, have shown a comparable MyD88-dependent regulatory mechanism in tolerant microglia, marked by the upregulation of negative regulators of TLR signaling [12,13,81]. This suggests that hypoxia not only modulates the inflammatory responses in BV-2 cells but also impinges on critical signaling pathways involved in the TLR4-signaling. While hypoxia appears to modulate the MyD88/NF-κB p65 pathway, further experiments are needed to establish a direct causal link between this pathway and LPS tolerance under hypoxic conditions.

Metabolic alterations serve as a key prerequisite in regulating the inflammatory responses of immune cells [82]. An increasing number of studies have shown that reduced levels of lactate, the end product of glycolysis, result in an anti-inflammatory (tolerant) phenotype, characterized by a diminished pro-inflammatory response [21,46,83]. In line with this, LPS-tolerant BV-2 cells exhibited reduced expression of key glycolytic genes, such as PFK1 and HK-2, accompanied by lower lactate production, which was more pronounced under hypoxic conditions. As demonstrated by others, glycolysis represents an early metabolic event actively involved in microglial activation, contributing to neuroinflammatory disorders [15,84]. This aligns with the emerging view that metabolic reprogramming, especially reduced glycolysis, which is typically associated with decreased pro-inflammatory reactions, is a crucial component of immune cell tolerance [21,83]. Similarly, our group’s data have previously reported a similar response, where LPS-tolerant murine primary microglia are characterized by decreased glycolytic capacity, as evidenced by reduced lactate levels and low extracellular acidification rates (ECAR) [46]. It is important to note also that ATP and its metabolites are key regulators of inflammatory processes, primarily by stimulating cytokine production and promoting the migration of immune cells [10,85]. These effects are largely mediated through the activation of purinergic receptors, specifically P2X and P2Y subtypes. Upon activation, these receptors trigger intracellular signaling cascades that lead to the release of pro-inflammatory cytokines such as TNF-α, IL-1β, and IL-6 [86,87]. Furthermore, ATP’s breakdown products, including adenosine, help modulate inflammation in a balanced manner. While ATP amplifies the inflammatory response, adenosine acts to dampen excessive inflammation by binding to A2A and A2B receptors, thereby providing a negative feedback mechanism that protects tissues from damage [88,89]. These findings underscore the importance of metabolic control, particularly glycolysis, shaping the inflammatory reactions in microglia, thereby providing potential avenues for modulating microglial activation and neuroinflammation in disease. Hypoxia, known to alter cellular metabolism, appears to further reinforce this metabolic shift in BV-2 microglia, enhancing their ability to maintain a stronger tolerant phenotype. In parallel, Abebayehu et al. revealed that declined inflammatory reactions are mediated through a HIF-1α-dependent suppression of microRNA (miR)-155 [90]. Consistent with this, a study showed that lactate mitigated neuronal injury by regulating microglial inflammation and promoting neuroprotective effects through HIF-1α following oxygen-glucose deprivation (OGD) [91].

Among their many functions, microglial migration and phagocytosis are critical functions in CNS that enable microglia not only to facilitate microglial surveillance but also influence the progression and resolution of neuroinflammatory responses [3,10,92]. LPS-tolerant BV-2 microglia exhibited increased migratory capacity, which was closely associated with the upregulation of specific markers (e.g., CD11a) and enzymes (e.g., MMP9) involved in cellular motility and tissue remodeling. As previously reported, sustained exposure to LPS, which induces a robust tolerance phenotype, is characterized by enhanced migratory capacity [21,93]. CD11a, a well-known integrin, regulates not only microglial adhesion to extracellular matrix components but also strongly controls their migration, facilitating movement through tissue [94,95]. Meanwhile, MMP9 plays a crucial role in breaking down the extracellular matrix, allowing microglia to traverse tissue barriers and reach damaged areas [96,97]. Microglial migration is often followed by phagocytosis as part of a coordinated response to neuroinflammation, a process essential for tissue repair, immune surveillance, and the resolution of inflammation. LPS-tolerant BV-2 microglia demonstrated increased phagocytic activity, which was associated with elevated expression of CD32, a surface receptor that mediates phagocytosis. Similarly, several studies on primary microglia have shown that induction of tolerance by LPS triggers increased phagocytic activity, which can alleviate cerebral β-amyloidosis in a mouse model of Alzheimer’s disease [13,98,99]. In neurodegenerative diseases, impaired or dysregulated phagocytosis by microglia has been implicated in the accumulation of toxic proteins and cellular debris, exacerbating disease progression [10,100]. Oxygen depletion under hypoxic conditions impaired both the migratory and phagocytic activities of BV-2 microglia, with these functions being closely regulated by the ERK 1/2 signaling pathway. The suppression of motility and phagocytosis under hypoxia suggests that oxygen deprivation critically modulates microglial dynamics, potentially impairing their ability to respond to injury or inflammatory cues in the brain microenvironment. ERK 1/2 activation triggers the phosphorylation of downstream targets involved in actin polymerization and microtubule dynamics, while also regulating the expression of phagocytic receptors [96,101,102,103]. Altogether, our data indicate that LPS-induced tolerance in microglia is not limited to suppressing excessive inflammatory responses, but also leads to increased migration and phagocytosis, facilitating tissue repair and clearance, thereby maintaining tissue integrity and restoring CNS homeostasis.

Long-term hypoxic conditions, characterized by sustained increases in HIF-1α, can induce a wide range of maladaptive cellular processes that contribute to chronic diseases, including neurodevelopmental impairments [104,105]. However, several reports indicate that short-term oxygen deprivation, referred to as intermittent hypoxia (IH), may exert favorable effects [106,107]. IH has been shown to promote adaptive responses in multiple organs, including the brain, where it can enhance neuroprotection, stimulate neurogenesis, increase resistance to injury, and improve neurological function [108,109,110]. Moreover, mild IH during pregnancy is not only well tolerated but also plays an essential role in fetal development and the initiation of labor by stimulating the synthesis and release of prostaglandins, regulating uterine contractility, and contributing to extracellular matrix remodeling—key processes required for cervical ripening and the onset of labor [111,112]. Altogether, we demonstrated that short-term hypoxia can enhance the immunological tolerance of microglial cells by regulating their inflammatory, metabolic, and functional activity. A finely tuned combination of immune tolerance and mild hypoxic conditions is essential for normal fetal development [113,114,115]. However, disruption of this balance through excessive inflammation or chronic pathological hypoxia may shift these adaptive mechanisms toward maladaptive outcomes with significant adverse health effects.

It is important to acknowledge the limitations of using immortalized cell lines, such as BV-2 microglial cells, when studying microglia, as they differ from primary microglial cells, which limits their physiological relevance and the extrapolation of findings to in vivo behavior. However, BV-2 cells, as an immortalized murine microglial cell line, represent a robust and widely used model for studying inflammatory and functional responses (e.g., phagocytosis), often exhibiting higher reactivity compared to other murine or human microglial cell lines, such as HMC3. Therefore, future studies using primary microglia or in vivo models are necessary, and these results should be interpreted with caution.

## 5. Conclusions

Overall, our findings indicate that short-term, mild hypoxic conditions enhance the development of tolerance-like phenotypes in BV-2 microglial cells, as evidenced by decreased expression of pro-inflammatory mediators and a concomitant reduction in glycolytic activity. At the functional level, while immune tolerance promotes increased migratory and phagocytic capacities, these effects are attenuated under hypoxia compared to normoxic conditions. These results highlight the critical interplay between metabolic reprogramming and immune regulation in microglia, suggesting that a controlled hypoxic microenvironment may contribute to the resolution of inflammation. By limiting excessive pro-inflammatory responses and promoting tissue surveillance and clearance functions, such conditions could support CNS tissue integrity and potentially aid in the restoration of homeostasis following injury or stress. Additional research using primary microglia or appropriate in vivo models is warranted to determine the physiological relevance of these in vitro findings.

## Figures and Tables

**Table 1:** Primers and their sequences used in this study.

Primer		Primer Sequences (5′–3′)
PFK1 (Phosphofructokinase 1)	Forward:	TGACATGACCATTGGCACAG
	Reverse:	TCTTGCTACTCAGGATTCGG
HK-2 (Hexokinase 2)	Forward:	ATTGTCCAGTGCATCGCGGA
	Reverse:	AGGTCAAACTCCTCTCGCCG
p65 (Transcription factor p65)	Forward:	CTTCCTCAGCCATGGTACCTCT
	Reverse:	CAAGTCTTCATCAGCATCAAACTG
CD11a (Integrin αL)	Forward:	AGATCGAGTCCGGACCCACAG
	Reverse:	GGCAGTGATAGAGGCCTCCCG
MMP9 (Matrix metallopeptidase 9)	Forward:	ACCACTAAAGGTCGCTCGGATGG
	Reverse:	AGTACTGCTTGCCCAGGAAGACG
CD32 (Cluster of differentiation 32)	Forward:	AATCCTGCCGTTCCTACTGATC
	Reverse:	GTGTCACCGTGTCTTCCTTGAG
GAPDH (Glyceraldehyde-3-phosphate dehydrogenase)	Forward:	CATGGCCTTCCGTGTTTCCTA
	Reverse:	CCTGCTTCACCACCTTCTTGAT

## Data Availability

The raw data supporting the conclusions of this article will be made available by the authors on request.

## Supplementary Materials

The following supporting information can be downloaded at: https://www.mdpi.com/article/10.3390/biology14111512/s1, Figure S1: Analysis of cell viability and cytotoxicity in LPS-primed BV-2 microglia under normoxic and hypoxic conditions in vitro; Figure S2: Western blot membrane of HIF-1α (132 kDa, Cat. No. sc-13515, Santa Cruz Biotechnology, Inc.) and β-actin (42 kDa, Cat. No. A5441, Sigma-Aldrich) protein; Figure S3: Western blot membrane of HIF-1α (132 kDa, Cat. No. sc-13515, Santa Cruz Biotechnology, Inc.) and β-actin (42 kDa, Cat. No. A5441, Sigma-Aldrich) protein; Figure S4: Western blot membrane of MyD88 (33 kDa, Cat. No. 4283, Cell Signaling) and β-actin (42 kDa, Cat. No. A5441, Sigma-Aldrich) protein; Figure S5: Western blot membrane of phospho-p44/42 MAPK (ERK1/2) (Thr202/Tyr204) (42/44 kDa, Cat. No. 9106, Cell Signaling), p44/42 MAPK (ERK1/2) (42/44 kDa, Cat. No. 9107, Cell Signaling), and β-actin (42 kDa, Cat. No. A5441, Sigma-Aldrich) protein; Figure S6: Western blot membrane of phospho-NF-κB p65 (65 kDa, Cat. No. 3033, Cell Signaling), and β-actin (42 kDa, Cat. No. A5441, Sigma-Aldrich) protein detected as previously described in the Section 2.5.

## Abbreviations

The following abbreviations are used in this manuscript:

ATP	Adenosine triphosphate
CD11a	Integrin alpha L
CD32	Cluster of differentiation 32
CNS	Central nervous system
DAMP	Damage-associated molecular pattern
ECAR	Extracellular acidification rate
ERK1/2	Extracellular signal-regulated kinase 1/2
GAPDH	Glyceraldehyde-3-phosphate dehydrogenase
HIF-1α	Hypoxia-inducible factor 1 alpha
HK-2	Hexokinase 2
HMC3	Human microglial clone 3
IFN-γ	Interferon gamma
IH	Intermittent hypoxia
IKK	IkappaB kinase
IL-6	Interleukin 6
LPS	Lipopolysaccharide
MAPK	Mitogen-activated protein kinase
MCP-1	Monocyte chemoattractant protein-1
miR	MicroRNA
MMP9	Matrix metalloproteinase 9
MyD88	Myeloid differentiation primary response gene 88
NF-κB	Nuclear factor kappa B
OGD	Oxygen-glucose deprivation
PAMP	Pathogen-associated molecular pattern
PaO_2_	Oxygen partial pressure
PFK1	Phosphofructokinase 1
PRRs	Pattern recognition receptors
ROS	Reactive oxygen species
TLR	Toll-like receptor
TNF-α	Tumor necrosis factor alpha
UP	Unprimed
US	Unstimulated
