# Supplementary material for: Hypoxia Supports LPS-Driven Tolerance and Functional Activation in BV-2 Microglial Cells
Source: Biology (Basel). 2025 Oct 28;14(11):1512. doi: 10.3390/biology14111512 (PMC12650609; doi:10.3390/biology14111512)
Supplement: Supplementary file 1 [file biology-14-01512-s001.zip › biology-3921417-supplementary.pdf]

Supplementary Figures

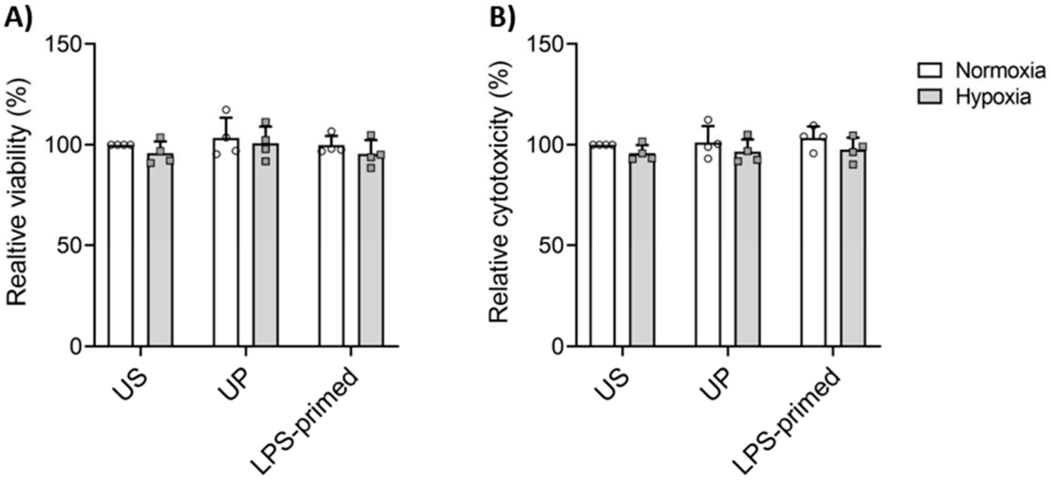

Figure S1. Analysis of cell viability and cytotoxicity in LPS-primed BV-2 microglia under normoxic and hypoxic conditions *in vitro*. BV-2 cells were stimulated accordingly to the two-step protocol as described. (**A**) Cell viability (N=4) performed by MTT assay and (**B**) cytotoxicity (N=4) by commercial kits under selected LPS concentration and hypoxic conditions was evaluated as described in the methods section. Results are depicted as scatter dot plots, mean + SD (unstimulated cells under normoxia assigned as 100%).

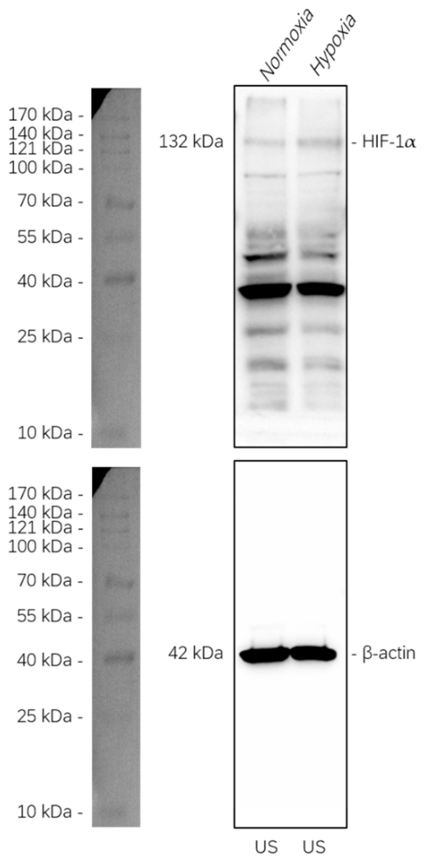

Figure S2. Western blot membrane of HIF-1α (132 kDa, Cat. No. sc-13515, Santa Cruz Biotechnology, Inc.) and β-actin (42 kDa, Cat. No. A5441, Sigma-Aldrich) protein detected as previously described in the Materials and Methods section (subsection *2.5. Protein extraction and SDS-PAGE Western Blotting*).

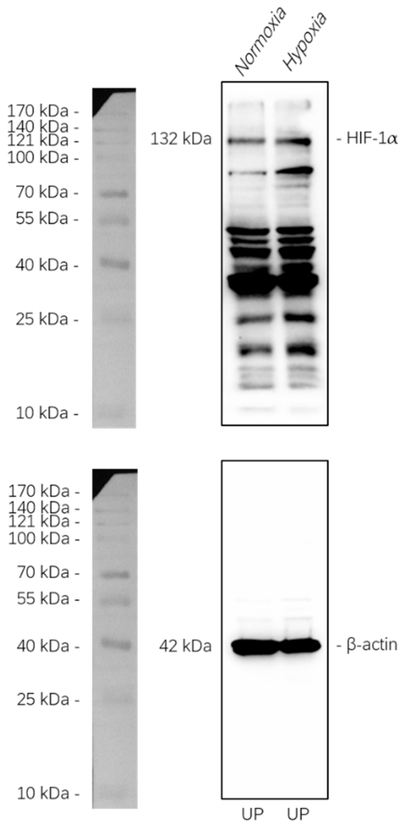

Figure S3. Western blot membrane of HIF-1α (132 kDa, Cat. No. sc-13515, Santa Cruz Biotechnology, Inc.) and β-actin (42 kDa, Cat. No. A5441, Sigma-Aldrich) protein detected as previously described in the Materials and Methods section (subsection *2.5. Protein extraction and SDS-PAGE Western Blotting*).

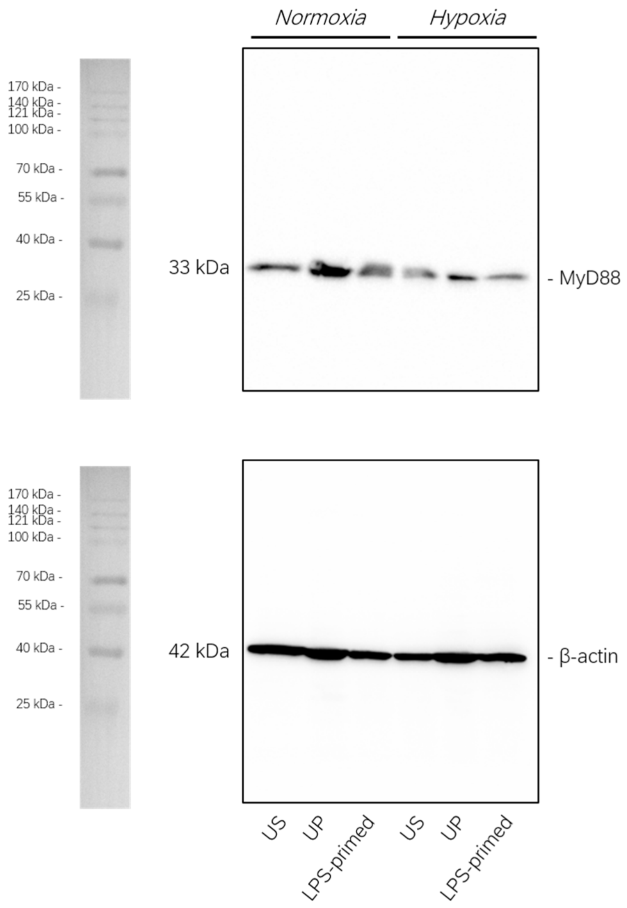

Figure S4. Western blot membrane of MyD88 (33 kDa, Cat. No. 4283, Cell Signaling) and β-actin (42 kDa, Cat. No. A5441, Sigma-Aldrich) protein detected as previously described in the Materials and Methods section (subsection *2.5. Protein extraction and SDS-PAGE Western Blotting*).

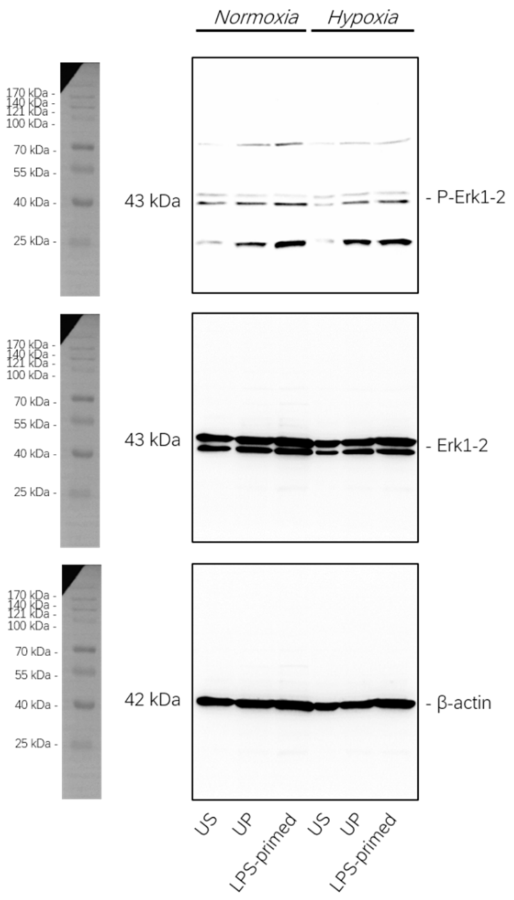

Figure S5. Western blot membrane of phospho-p44/42 MAPK (ERK1/2) (Thr202/Tyr204) (42/44 kDa, Cat. No. 9106, Cell Signaling), p44/42 MAPK (ERK1/2) (42/44 kDa, Cat. No. 9107, Cell Signaling), and β-actin (42 kDa, Cat. No. A5441, Sigma-Aldrich) protein detected as previously described in the Materials and Methods section (subsection *2.5. Protein extraction and SDS-PAGE Western Blotting*).

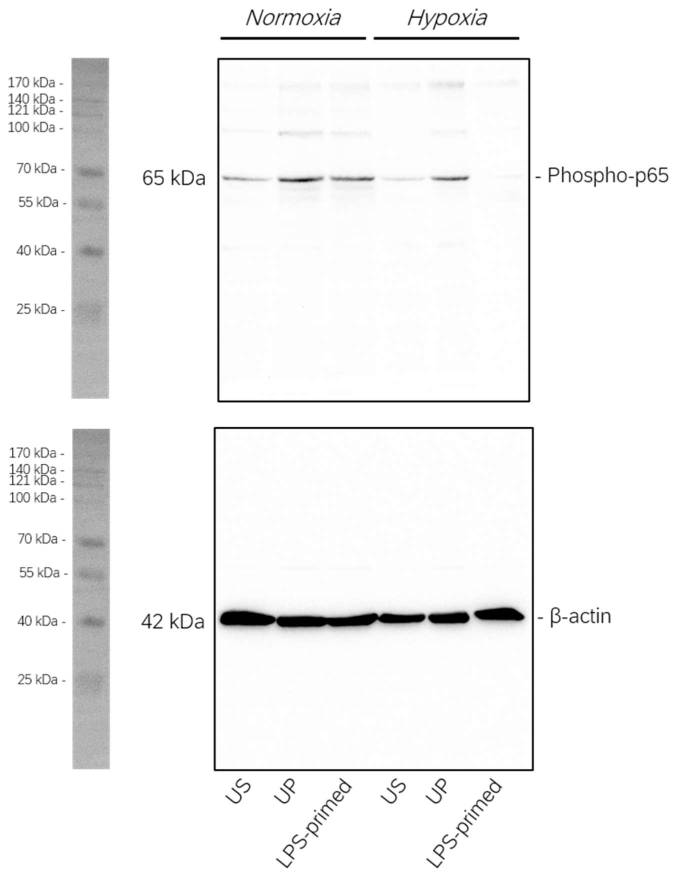

Figure S6. Western blot membrane of phospho-NF-κB p65 (65 kDa, Cat. No. 3033, Cell Signaling), and β-actin (42 kDa, Cat. No. A5441, Sigma-Aldrich) protein detected as previously described in the Materials and Methods section (subsection *2.5. Protein extraction and SDS-PAGE Western Blotting*).
